# Abnormal prostate microbiota composition is associated with experimental autoimmune prostatitis complicated with depression in rats

**DOI:** 10.3389/fcimb.2022.966004

**Published:** 2022-09-30

**Authors:** Feng Liu, Xiaolin Xu, Zhong Wang, Peng Wu

**Affiliations:** ^1^ Department of Urology, Nanfang Hospital, Southern Medical University, Guangzhou, China; ^2^ Department of Urology, Affiliated Sixth People’s Hospital South Campus, Shanghai Jiao Tong University, Shanghai, China

**Keywords:** prostatitis, depression, prostate microbiota, inflammation, metabolic phenotype

## Abstract

**Background:**

Microbiota play essential roles in the pathogenesis of prostatitis and depression. However, the changes in prostate microbiota have not yet been explored in rats with prostatitis/depression. This study aimed to investigate the changes of prostate microbiota in rats with prostatitis/depression.

**Methods:**

Rats with experimental autoimmune prostatitis (EAP) complicated with depression were constructed through injection of rat prostate antigen with immunoadjuvants followed by application of chronic unpredictable mild stress (CUMS). The rats were subjected to inflammatory factor detection and behavioral testing to confirm the establishment of the model. Subsequently, the prostate microbiota was assayed in the rats and compared by 16S rRNA gene sequencing.

**Results:**

A rat model of EAP complicated with depression was established and confirmed by increases in IL-1β, IL-6, and TNF-α as well as the occurrence of depressive‐like behaviors. EAP/CUMS significantly altered the richness, evenness, and composition of prostate microbiota. Forty-six taxonomic biomarkers for prostate microbiota were enriched in rats with EAP/depression and exhibited statistically significant and biologically consistent differences. Metabolomics profiling revealed that EAP/depression was associated with reductive acetyl coenzyme A pathway, L-lysine fermentation to acetate and butanoate, protein N-glycosylation and purine nucleobases degradation I, which is regulated by *DCE29, Nocardioes, Helicobacter* and *Dorea*.

**Conclusion:**

Findings from the study demonstrate the existence of abnormal prostate microbiota in EAP complicated with depression and may be helpful in the treatment of comorbid diseases of prostatitis and depression.

## Introduction

Prostatitis is the most common inflammation in adult men. Chronic prostatitis/chronic pelvic pain syndrome (CP/CPPS) (NIH-III) comprise the highest proportion of cases of prostatitis, accounting for 90%-95% of prostatitis (Hu et al., 2019). The clinical etiology of CP/CPPS is complex—the symptoms are changeable, the course of disease is prolonged, the recurrence rate is high, and it leads to sexual and mental disorders that can seriously affect the quality of life of patients. The incidence of depression, anxiety, and other low mood conditions among patients with CP in China is as high as 45% (Guan et al., 2015). Studies have shown that a depressive personality can induce early prostatitis symptoms, and patients with prostatitis are also prone to psychological problems (Chung et al., 2011; Lien et al., 2020). In recent years, the increasing incidence of CP/CPPS complicated with depression has led to some patients committing suicide, which subsequently has a marked negative impact on the health of men. Therefore, the mechanism of prostatitis complicated with depression is important.

Emerging studies indicate that prostate is found to be a reservoir of many bacterial communities (Menon et al., 2013; Bajic et al., 2019). The diversity of microbiota in the prostate of patients with CP/CPPS is significantly different to that of healthy people (Nickel et al., 2015; Shoskes et al., 2016). For example, Kogan et al. (Kogan et al., 2021) reported that in patients with chronic bacterial prostatitis, a predominance of anaerobes or a combination of aerobes and anaerobes in a titer of ≥103 colony-forming units per mL in post-massage urine is associated with worse clinical status. At present, accumulating evidence confirm that depression is closely related with the health condition of the brain-gut axis, and maintaining/restoring the normal condition of gut microbiota helps in the prevention/therapy of mental disorders. Interestingly, Du’s study (Du et al., 2020) has provided evidences that abnormal gut microbiota composition is associated with experimental autoimmune prostatitis‐induced depressive‐like behaviors in mice. However, the changes of prostate microbiota in prostatitis complicated with depression have not yet been explored.

In this study, a rat model with EAP/depression was established and the prostate microbiota and metabolic profile were analyzed to determine whether bacterial genera and specific metabolic phenotypes are associated with prostatitis complicated with depression. To our knowledge, this is the first study exploring the difference of prostate microbiota in prostatitis complicated with depression.

## Materials and methods

### Animals

Male Wistar rats (weight 180-200 g) were purchased from Shanghai Jiao Tong University [SCXK (shanghai) 2018-0007] and housed two per cage under a constant temperature (22 ± 0.5°C) and a 12-h light/dark cycle with free access to food and water. Rats were randomly divided into three groups: NC group (n=15), experimental autoimmune prostatitis (EAP) group (n=15), and EAP+chronic unpredictable mild stress (CUMS) group (n=15). EAP or/and depression rat models were constructed after one week adapting to the new environment. All animal studies and experiments were approved by the Institutional Animal Care and Use Committee of Shanghai Jiao Tong University.

### Purification of prostatic steroid-binding protein

Purification of PSBP was conducted as previously reported (Chen et al., 2019). Briefly, prostate tissues were lysed in lysis buffer (0.5% TritonX-100, 0.09% NaCl, and protease inhibitor cocktail), centrifuged at 12,000 *g* for 20 min, and the supernatant was collected. The concentration of protein in the supernatant was detected by BCA kit (cat. ab102536, Abcam, England), adjusted to 40 mg/mL by protein extract solution, and then stored at −80°C for use in later experiments.

### Rat model of EAP

For the first immunization, PSBP was emulsified at 1:1 with complete Freund’s adjuvant (Sigma, USA). The control (NC) group was injected with 0.1 mL saline solution in the dorsal lobe of the left prostate and abdominal cavity, while 0.1 mL emulsified PSBP was injected into the dorsal lobe of the left prostate of rats in the EAP groups and EAP+CUMS groups, and 0.1 mL PBSP was injected intraperitoneally. After operation, all rats were intramuscularly injected with penicillin at a dose of 300,000 U/kg body weight once a day for 7 days to prevent infection.

Four weeks after the first immunization, a booster immunization of PSBP emulsified with incomplete Freund’s adjuvant was subcutaneously injected into the 3 points of ventral and dorsal of the rats, and each point was 0.05~0.1 mL.

### Chronic unpredictable mild stress procedure

Depression-like behaviors of the rats were induced by CUMS as previously described (Huang et al., 2019). Ten different stress factors were used: 1, fasting food and water; 2, swimming in 25°C water for 20-30 min; 3, inclining squirrel cage 45° for 12-24 h; 4, wet cage for 12-24 h; 5, stroboflash at night with 120 flashes/min for 6 h; 6, reversal of the 12-h light/dark cycle; 7, paired feeding; 8, putting the rats into a retainer (20 cm × 9 cm) for 2-4 h; 9, 2-h alternation of light and dark; and 10, noise for 30 min in a random order. Rats were exposed to one stress factor at a different time point every day.

### Hematoxylin and eosin staining

HE staining was performed as described previously (Xiao et al., 2020). Briefly, hippocampus and prostate tissues were fixed with 4% paraformaldehyde at 4°C overnight and then embedded in paraffin. Hippocampus and prostate slices (5 μm) were stained with hematoxylin solution for 5 min after deparaffinization and rehydration. The slices were then soaked in HCl-ethanol five times, rinsed with distilled water, and stained with eosin for 3 min. Slices were washed again with distilled water, then dehydrated with graded alcohol and cleared with xylene before mounting with neutral balsam for light microscope observations.

### Cytokine measurements

Prostate tissue samples were collected from rats in each group and the supernatant fluid of the samples was harvested after centrifugation at 800 g for 20 min. ELISA kits (Westang, China) were used to detect the concentration of TGF-β (cat. F3766), IL-6 (cat. F3743), and IL-1β (cat. F3739).

### Open field test

OFTs were performed as previously described (Yang et al., 2018). Briefly, rats were placed in the center of an open field reaction box (100 cm × 100 cm × 40 cm) in a quiet room for 6 min. There was a video camera 2 m above the box to track the movement of the rats. Spontaneous alternation performance (the number of squares crossed, movement distance, and movement speed) was assessed with the SMART 3.0 video tracking system (Harvard Apparatus, USA).

### Forced swimming test

FST is an important index to evaluate the depressive-like behavior of rats (Yang et al., 2018), and was performed as described previously (Yang et al., 2018). Briefly, test rats were placed in a cylinder containing 25°C water (30 cm diameter, 50 cm height and 35 cm water depth) 24 h before the formal experiment. The suspended limbs of the rats could not touch the bottom of the cylinder. After 15 min, the rats were removed, dried, and returned to their cages. The FST for 6 min was conducted the following day under identical conditions. The immobility time and struggle time of the rats in the 6 min were recorded. Immobility time was defined when only the head of the rat was out of the water and the body was floating in the water with the limbs moving slightly but not struggling.

### Sucrose preference test

The SPT was used to evaluate the degree of loss of pleasure, which is the core symptom of depression in animals and therefore indicative of the success of an animal model of depression (Huang et al., 2017; Yang et al., 2018). SPT was performed described as previously (Yang et al., 2018). Briefly, rats were housed in individual cages and acclimatized to drinking two bottles of water for 48 h, then two bottles of 1% sucrose for 48 h. Water was then withdrawn for 24 h, and the rats were subsequently exposed to a bottle of 1% sucrose and a bottle of water for 2 h in the dark phase, with the bottle positions switched after 1 h (for 2 h test). The total consumption of water and sucrose was measured, and sucrose preference was calculated using the following formula: Sucrose preference (%) = (Sucrose consumption)/(Total consumption of water and sucrose) * 100%.

### 16S rRNA gene sequencing

The rats were sacrificed, perfused with normal saline, and the left ventral lobe of the prostate was stripped under sterile conditions. Total DNA from prostate tissue samples of rats was extracted by DNeasy@Blood&Tissue kit (cat.69504, Qiagen, China) and the quality of DNA was detected by spectrophotometer. Universal primers for the V4 region of the bacterial 16S rRNA gene were used for PCR amplification and high-throughput sequencing on the Illumina MiSeq platform. Sequencing data were processed and analyzed by Personalbio Technology Company (Shanghai, China). The original high-throughput sequencing data were screened according to the sequence quality, and problem samples were retested and/or subjected to supplementary testing. The sequences were then divided into libraries and samples according to index and Barcode information, and the barcode sequence was removed.

### Analyses of prostate microbiota

QIIME2 was used to obtain the operational taxonomic units (OTUs). Due to the distribution of ASV/OTUs in different samples, the α-diversity level of each sample was evaluated, and the sparse curve was used to reflect whether the sequencing depth was appropriate. Based on the results of ASV/OTU analysis, the microbial diversity and β-diversity of the samples were analyzed. A matrix was obtained, and principal coordinate analysis and 3D visualization were conducted using R software based on the Bray-Curtis distance. Kruskal-Wallis and Wilcoxon tests were used to identify significantly different bacteria (biomarkers), and linear discriminant analysis was used to evaluate the influence of these bacteria. Observed species and Chaol indices were used to indicate species richness, while Shannon and Simpson indices were used to estimate community diversity. β-diversity analysis was used to evaluate differences in species complexity among samples. β-diversity on weighted UniFrac were calculated using QIIME (Version 1.9.1). The hierarchical clustering analysis was performed using the unweighted pair group method with arithmetic mean. Furthermore, LEfSe (Linear Discriminant Analysis Effect Size) software was used to compare species differences among groups, and Linear Discriminant Analysis (LDA) was used to find the different intestinal bacteria among groups (LDA Score >3.19). Using the abundance data of ASV/OTUs, the ASV/OTUs with a total number of sequences less than 10 and number of samples less than 5 were filtered and removed. Using the ASV/OTU table not flattened, the “classify_samples_ncv” function was employed for random forest analysis and nested hierarchical cross test. The sparcc algorithm was used to construct the correlation matrix, the filtering threshold of correlation value was determined using the random matrix theory, and the correlation network data were constructed with iGraph.

### Analyses of metabolic phenotypes

Pathway enrichment analysis of differential metabolites was performed using the Kyoto Encyclopedia of Genes and Genomes (KEGG) database, MetaCyc data, and Clusters of Orthologous Groups (COG) data. After obtaining the abundance data of metabolic pathways, the metanomeseq method was used to identify the metabolic pathways with significant differences between groups. According to the selected pathway, the corresponding data in the hierarchical sample metabolic pathway abundance table were used to obtain the species composition of the metabolic pathway.

### Statistical analysis

Data were expressed as the mean ± standard deviation (SD). GraphPad Prism software (Graphpad Software, San Diego, CA, USA) was used for statistical analysis. Statistical analyses of multiple group comparisons were performed using Kruskal-Wallis H tests. *P<0.05 and **P<0.01 were considered statistically significant differences.

## Results

### Establishment of a rat model with EAP and depression

**Figure 1:**
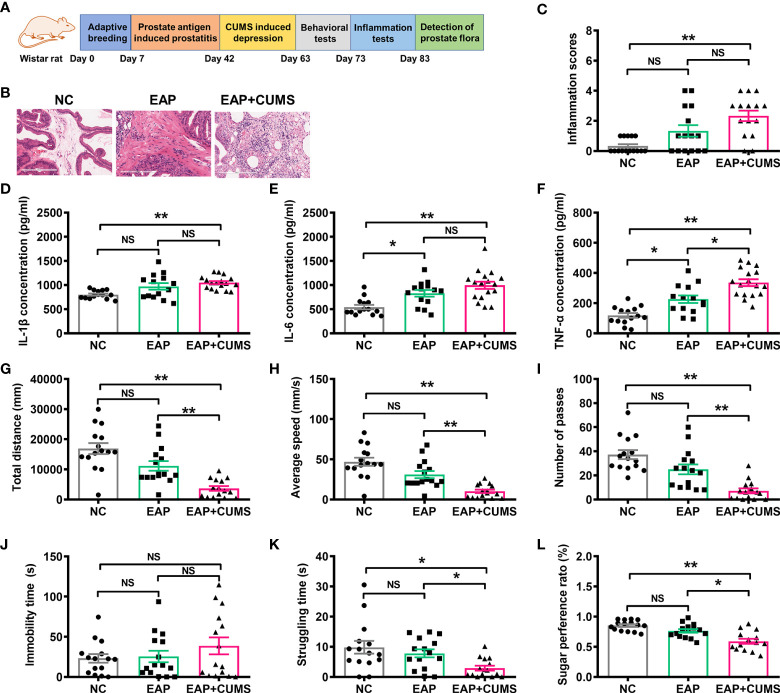
Induction of rat model with EAP/depression. **(A)** Schematic of the experimental design in this study. **(B, C)** Representative images of H&E staining of prostate tissue. Concentration of IL-1β **(D)**, IL-6 **(E)**, and TNF-α **(F)** in serum of rats with EAP or/and depression determined by ELISA. The detection of behaviors in rats with EAP or/and depression in open field test (OFT), including total distance **(G)**, and average speed **(H)**, and number of passes **(I)**. Effects of EAP or/and depression on rats subjected to the forced swim test (FST), including immobility **(J)** and struggling time **(K)**. **(L)** Sugar preference ratio of rats with EAP or/and depression in sucrose preference test (SPT). ns, P>0.05, * P<0.05, ** P<0.01.

Based on previously described studies (Chen et al., 2019), a PSBP-induced EAP and CUMS-induced depressive‐like state was established in male Wistar rats. The experimental paradigm is shown in **Figure 1A**. To confirm the effect of PSBP and CUMS on the prostate tissues of rats, H&E staining was performed and histological changes in the prostate tissues were analyzed. The morphology of glandular epithelium and stroma was normal, and there was no inflammatory cell infiltration in the NC group. However, in EAP and EAP+CUMS groups, the prostatic stroma was hyperemic with edema present, and there were numerous inflammatory cells around the glandular cavity. Compared with the NC group, significant pathological changes were observed in EAP and EAP+CUMS groups (**Figures 1B, C**). Subsequently, inflammatory factors, including, IL-1β, IL-6 and TNF-α, in the prostate tissue were measured by ELISA. Significant increases in IL-1β, IL-6, and TNF-α were detected in the EAP+CUMS group compared with the NC group (all P<0.01); meanwhile, in addition, the EAP group had obvious influence on IL-6 and TNF-α compared with the NC group (all P<0.05) (**Figures 1D–F**). Next, OFT, FST, and SPT were conducted to explore the depressive-like behaviors of the rats. In the OFT, decreases in total distance, average speed, and number of passes were noted in the EAP+CUMS group compared with the NC group (all P<0.01), while CUMS exposure significantly reduced total distance, average speed, and the number of passes compared with the EAP group (all P<0.01) (**Figures 1G–I**). There was no significant difference in immobility time among all three groups in the FST (all P>0.05), while there was significant difference in struggling time between EAP+CUMS group and EAP group and between EAP group and NC group (all P<0.05, **Figures 1J, K**). In addition, the SPT revealed that there was no obvious change in sugar preference in the EAP group compared with the NC group (P>0.05), but CUMS exposure led to a significant decrease in sugar preference ratio compared with the EAP group (P<0.01) (**Figure 1L**). These findings verified that the models of prostatitis and depression had been successfully established.

### Prostate antigen/CUMS exposure induces prostatic microbial dysbiosis in rats

**Figure 2:**
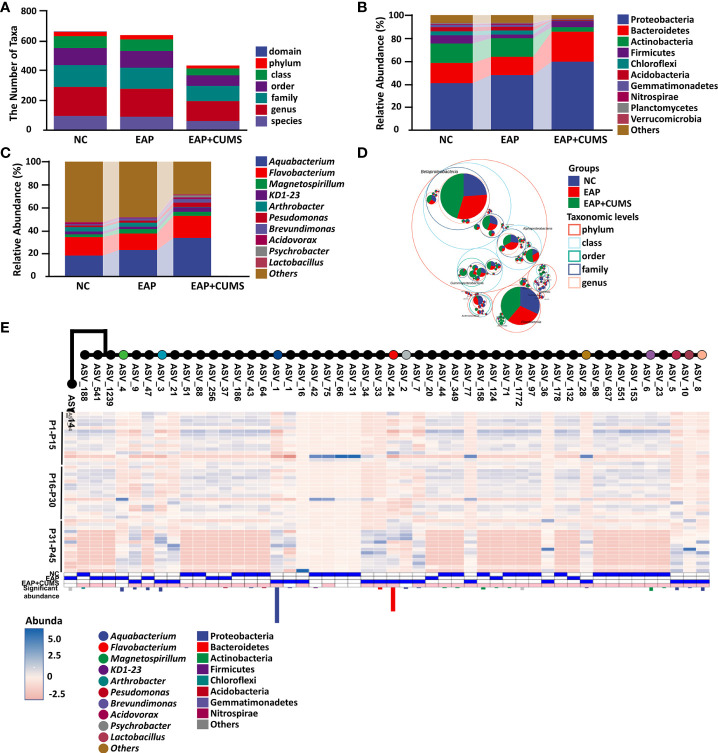
EAP or/and CUMS-induced prostatic microbial dysbiosis in rats. **(A)** Number of taxa of prostate microbiota of different samples in the three groups (NC, EAP, and EAP+CUMS). **(B)** Relative abundance of prostate microbiota at phylum level in the three groups. **(C)** Relative abundance of prostate microbiota at genus level in the three groups. **(D)** Taxonomic tree in packed circles from the three groups for prostate microbiota. The innermost dots represent the ASV/OTUs of the top 100 abundances, and the area is proportional to the abundance of the ASV/OTUs. Additionally, the larger the sector area, the higher the abundance of this taxon in the corresponding grouping. **(E)** Phylogenetic tree plot of prostate microbiota at the taxa level in different groups.

To explore the changes of prostate microbiota in EAP/depression rats, high-throughput detection and bioinformatic analysis of prostate microbiota in different prostate samples of rats from different groups was conducted. After quality control, denoising, and chimera removal, samples were rarefied to an even sampling depth of 155,344 reads for prostate microbiota. The number of taxa and relative abundance of prostate microbiota in different samples are shown in **Figures 2A–C**. CUMS exposure resulted in a significant decrease in abundance of prostate microbiota (**Figure 2A**). At the phylum level, Proteobacteria was the predominant phylum in the prostate microbiota of the rats, with a total abundance of nearly 41% in the NC group, 48% in the EAP group, and 60% in the EAP+CUMS group, respectively, followed by the phyla Bacteroidetes, Actinobacteria, and Firmicutes. There were significant decreases in the relative abundances of Chloroflexi, Acidobacteria, Gemmatimonadetes, Nitrospiraeand, and Planctomycetes, and significantly increased relative abundances of Proteobacteria and Bacteroidetes in the EAP+CUMS group (**Figure 2B**). At the genus level, *Aquabacterium* was predominant in the prostate microbiota of the rats, with a total abundance of approximately 17.58% in the NC group, 22.54% in the EAP group, and 33.11% in the EAP+CUMS group, respectively, followed by the genera *Flavobacterium*, *Magnetospirillum*, *Arthrobacter*, *Pseudomonas*, *Brevundimonas*, *Acidovorax*, *Psychrobacter*, and *Lactobacillus*. Significantly decreased relative abundances of the genus *Arthrobacter*, and significantly increased relative abundances of the genera *Aquabacterium*, *Flavobacterium*, *Magnetospirillum*, KD1-23, *Pseudomonas*, *Brevundimonas*, *Acidovorax*, *Psychrobacter*, and *Lactobacillus* were detected in the EAP+CUMS group (**Figure 2C**). The phylogenetic tree plot and taxonomic tree showed similar results (**Figures 2D, E**). These findings suggested that EAP or/and depression induces prostatic microbial dysbiosis.

### CUMS exposure changes α- and β-diversity of prostate microbiota in EAP rats

**Figure 3:**
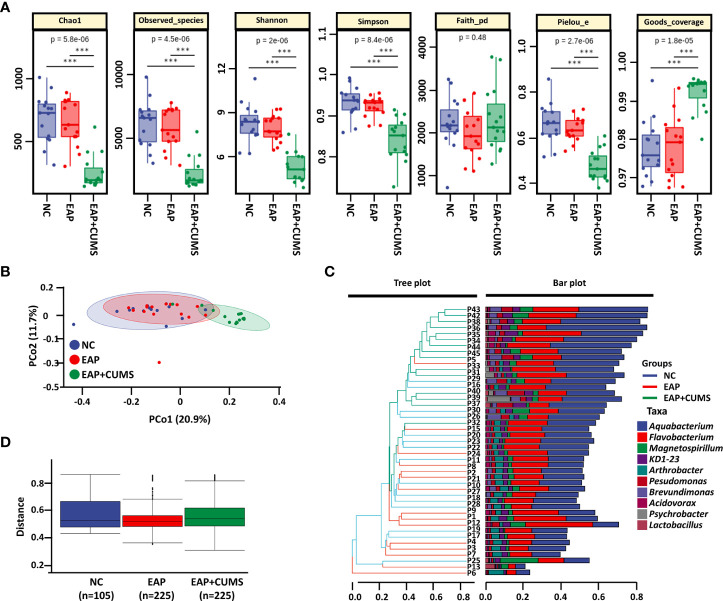
EAP or/and CUMS changes the diversity of prostate microbiota. **(A)** α-diversity analysis of prostate microbiota by Chao1, observed-species, Shannon, Simpson, faith-pd, pielou-e, and goods-coverage indices. **(B)** PCoA plot of β-diversity based on Jaccard and Bray-Curtis analysis in the three groups (NC, EAP, and EAP+CUMS) for prostate microbiota. **(C)** Hierarchical clustering analysis of β-diversity in different samples of the three groups. **(D)** Permutational multivariate analysis of variance of β-diversity of prostate microbiota. *** P<0.001.

α- and β-diversity indices are often used as indicators for comprehensive evaluation of overall species diversity. For α-diversity in the current study, significant differences in prostate microbiota were present among the three groups of rats by the indices of Chao1, observed-species, Shannon, Simpson, pielou-e, and Goods-coverage, but not faith-pd (**Figure 3A**). However, no significant differences in prostate microbiota were found between the NC group and the EAP group using Chao1, observed-species, Shannon, Simpson, faith-pd, pielou-e, and Goods-coverage indices (all P <0.05). For β-diversity analysis, the PCoA plot demonstrated that EAP did not lead to changes in the structure of prostate microbiota, but rats with EAP/depression possessed a distinct structure of prostate microbiota compared with EAP rats (**Figure 3B**). Moreover, hierarchical clustering analysis of prostate microbiota revealed that most of the NC samples, EAP samples, and EAP+CUMS samples clustered in their own groups (**Figure 3C**). Furthermore, inter-group difference analysis showed that there were significant differences among the three groups (**Figure 3D**). These results indicated that EAP or/and depression changes the diversity of the prostate microbiota.

### Analysis of species difference and marker species of prostate microbiota

**Figure 4:**
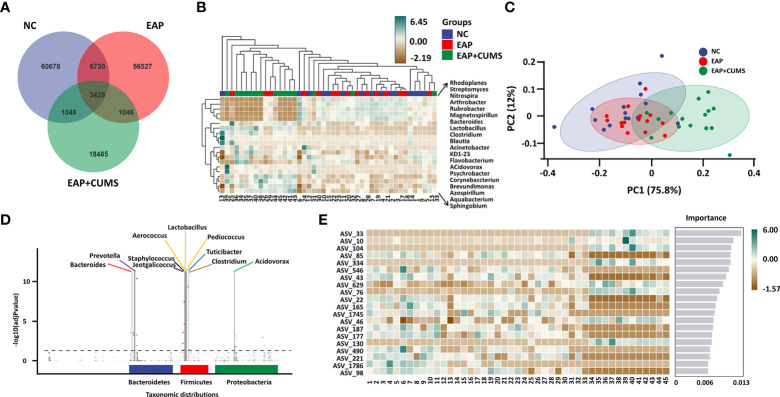
Key taxa of prostate microbiota among the experimental three groups (NC, EAP, and EAP+CUMS). **(A)** Venn diagram showing the number of different microbiota among the three groups. **(B)** Heatmap showing differences in species composition of prostate microbiota among samples of the three groups. **(C)** PCA analysis of species composition of prostate microbiota in different groups **(D)**. MetagenomeSeq analysis of taxonomic distributions of prostate microbiota. Each dot or circle in the coordinate system represents one ASV/OTUs, and the size represents its relative abundance. The dotted line separates the significant difference from the insignificant ASV/OTUs. The points with significant difference are marked by colored dots or circles, and the points with insignificant difference are represented by gray circles. **(E)** PLS-DA analysis of marker species of prostate microbiota in the three groups. NC group: P1-P15; EAP group: P16-P30; EAP+CUMS group: P31-P45;.

**Figure 5:**
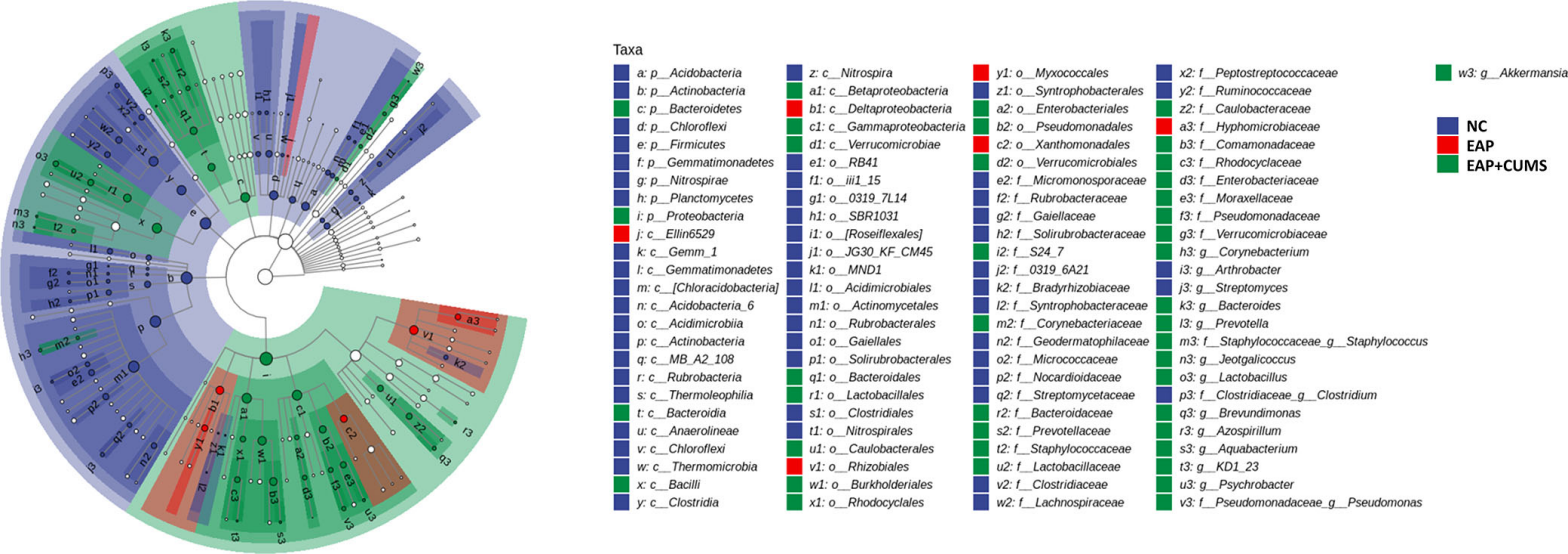
LDA and LEfSe analyses the key taxa of prostate microbiota among different groups. The taxonomic branch diagram shows the taxonomic hierarchy of the main taxa from phylum to genus (from inner circle to outer circle) in the sample community. The node size corresponds to the average relative abundance of the taxon; Hollow nodes represent taxa with no significant difference between groups, while nodes with other colors (such as green and red) indicate that these taxa reflect significant differences between groups and have high abundance in the grouped samples represented by this color. Letters identify the names of taxa with significant differences between groups.

To identify the unique species among the different groups of rats, a Venn diagram for prostate microbiota was plotted. A total of 3,428 microbiota co-existed in the three groups, while there were 60,678, 56,527, and 18,465 unique microbiota in the NC, EAP, and EAP+CUMS groups, respectively (**Figure 4A**). To further compare differences in species composition among the three groups and show the distribution trend of species abundance for each sample, the abundance data of genera in the top 20 of average abundance were used to draw hierarchical clustering analysis for species composition analysis. The species composition of the prostate microbiota was different in the EAP+CUMS group compared with that of the NC group or the EAP group, but was similar in the EAP and NC groups (**Figure 4B**). Similar results were also obtained from the PCA plot (**Figure 4C**). The differences in species composition among groups did not mean that there were differences in the components of all species, but often the differences were in the distribution of some components. MetagenomeSeq analysis revealed that there were increases in the adjPvalue of Bacteroidetes (*Bacteroides*, *Prevotella*, and *Bacteroides*), Firmicutes (*Lactobacillus*, *Jeotgalicoccus*, *Lactobacillus*, *Turicibacter*, *Clostridium*, *Aerococcus*, *Staphylococcus*, and *Pediococcus*) and Proteobacteria (*Rubrivivax*, *Pelomonas*, *Aquabacterium*, *Acidovorax*, and *Helicobacter*) in the EAP+CUMS group compared with the EAP group (**Figure 4D**). To identify biomarkers in the different groups of rats, LEfSe and LDA analysis were performed and demonstrated that 101 taxa were distinguishing for prostate microbiota in the three groups: 49 for the NC group, six for the EAP group, and 46 for the EAP+CUMS group. At the genus level, there were three prostatitis-related genera in the prostate, which were *Arthrobacter*, *Streptomyces*, and *Clostridium*, while 12 genera characterized the EAP+CUMS group, especially *Aquabacterium*, *Brevundimonas*, and *Pseudomonas* (**Figure 5**). Random Forests displayed the importance of species in EAP/depression; the top five were ASV_33, ASV_10, ASV_104, ASV_85, and ASV_334 (**Figure 4E**).

### EAP/depression rats exhibit a significantly altered metabolic profile

**Figure 6:**
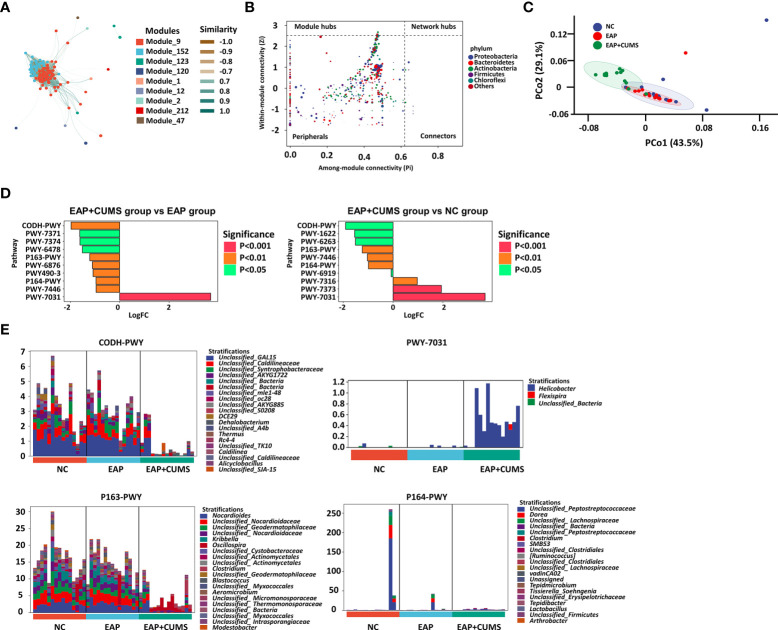
The metabolite profile related to EAP or/and depression. **(A)** Construction of associate network of prostate microbiota. The node represents the ASV/OTUs in the sample; The node size is in direct proportion to its abundance; The inter node connection indicates that there is a correlation between the two connected nodes. **(B)** ZIPI analysis for keystone species of prostate microbiota in the three groups. The node size was positively correlated with its abundance, and the nodes belonging to the top 5 taxa with the highest abundance were identified by different colors. **(C)** PCoA plot analysis of cell function for prostate microbiota among the three groups. **(D)** Metabolic pathway difference analysis for prostate microbiota. **(E)** Relative species composition of cell metabolism for prostate microbiota.

The similarity between the nine modules of prostate microbiota communities in the three experimental groups were analyzed by associated network analysis based on random matrix theory (**Figure 6A**). The EAP+CUMS group had a significant positive correlation with nodes with the highest abundance of the first five taxa were Firmicutes, Bacteroidetes, Proteobacteria, Tenericutes and Actinobacteria in intestine (**Figure 6B**). To better explain the function of these significantly changed microbiota, the metabolic phenotypes were investigated. PCo2 analysis by Bray-Curtis distance showed that the EAP+CUMS group possessed a distinct metabolic phenotype (**Figure 6C**).

Compared with the EAP group, the EAP/CUMS group promoted one metabolic pathway and inhibited nine metabolic pathways (**Figure 6D**). Furthermore, compared with the NC group, the EAP/CUMS group promoted three metabolic pathways and inhibited seven metabolic pathways (**Figure 6D**). Interestingly, there was four metabolic pathways including CODH-PWY (reductive acetyl coenzyme A pathway), P163-PWY (L-lysine fermentation to acetate and butanoate), PWY-7031 (protein N-glycosylation (bacterial)) and P164-PWY (purine nucleobases degradation I (anaerobic)), which was involved in EAP complicated with depression. The genera, including *DCE29, Nocardioes*, *Helicobacter* and *Dorea* encoded these genes involved in these different metabolic pathways (**Figure 6E**).

## Discussion

Microbial disorders can cause prostatitis and prostate disease is associated with alterations of abundance and diversity of gut microbiota and intestinal metabonomics (Li et al., 2022). Interestingly, gut microbiota can cause pathological changes in microbiota microenvironments through the microbiota-gut-brain axis, even destroying the activity and function of the central nervous system, and directly resulting in the occurrence of Parkinson’s Disease (PD), Alzheimer’s Disease (AD), and depression (Caputi and Giron, 2018; Liang et al., 2018; Megur et al., 2020). CP/CPPS is a very common disease in adult men, often accompanied by fatigue, pain and depression (Hu et al., 2019). The incidence of depression among patients with CP/CPPS in different regions of the world is 14.4-45% (Guan et al., 2015; Baden et al., 2020). However, the changes of microbiota on prostatitis complicated with depression have yet to be elucidated. Based on the reported studies (Du et al., 2019; Xiang et al., 2020), a rat model with EAP and depression was constructed in the current study to explore the changes of prostate microbiota. The prostatic stroma was hyperemic with edema present, and there were numerous inflammatory cells around the glandular cavity in the rates of the EAP and EAP+CUMS groups, which was congruent with previous reports (Chen et al., 2019). Proinflammatory cytokines are closely linked to the occurrence and development of CP/CPPS and depression (Wang et al., 2018; Peng et al., 2020). Therefore, to confirm the occurrence of inflammation in the EAP rat model, proinflammatory cytokines in the prostate of the rats were detected. As expected, proinflammatory cytokines such as IL-1 β, IL-6, and TNF- α were significantly increased in the EAP and EAP+CUMS rats. Additionally, in the current study, depression-like behaviors were induced by CUMS in EAP rats, as indicated by the results of OFT, FST, and SPT experiments. These findings proved that the models of EAP complicated with depression had been successfully established for the study of prostatic microflora. However, there was no significant difference in depression like behavior in EAP group. It may be due to the not high probability of depression like behavior in prostatitis rats and the limited number of rat used in the study.

As an important part of the urinary tract, the prostate has an excretory tube connected with the urethra, thus it is not a sterile organ and its microbiota characteristics vary with the change of pathological state (Wu et al., 2017). There is accumulating evidence that the pathological state of the prostate may lead to changes in antibacterial components in the prostatic fluid, leading to the growth of prostate microbiota and stimulating inflammation (Porter et al., 2018). Therefore, changes in the prostate microbiota of EAP/depression rats were explored in the current study. To our knowledge, this is the first study to use 16S rRNA gene sequencing to investigate the changes in prostate microbiota and the metabolic profile of these bacteria. EAP or/and depression had significant effects on the richness, evenness, and composition of prostate microbiota, especially Proteobacteria, Bacteroidetes, Actinobacteria, and Firmicutes. This differed from the report by Geng et al., which did not observe any significant difference between the two groups at the phylum level: Bacteroidetes, Firmicutes, and Proteobacteria represented approximately 98% of the total microbiota (Geng et al., 2019). We speculate that CUMS may lead to alterations in the prostate environment in which microorganisms live as well as the difference of environment between prostate and intestine leading to the alterations at the phylum level.

To further explain the specific microbiota-related changes in EAP complicated with depression, the diversity within and between habitats for gut and prostate microbiota were investigated. In the present study, clear evidence is provided that the α- and β-diversity of prostate microbiota were significantly higher compared with those of the control group. However, there was no significant difference in α- and β-diversity between the EAP group and the NC group, which was different from the studies of Tian et al. (Tian et al., 2020) and Geng et al. (Geng et al., 2019). Depression may dominate the effect on the prostate microbiota. In addition, the major differential species belonged to the phyla Bacteroidetes, Firmicutes, and Proteobacteria in the current study, suggesting that these microflora are important participants in human health. Growing evidence has indicated that changes in the composition of microflora may lead to the transformation of microflora and activation of the immune system, and eventually to the development of inflammatory diseases (Balato et al., 2019). Demmer et al. (Demmer et al., 2017) reported that Bacteroidetes and Firmicutes are positively associated with inflammation, whereas Proteobacteria are inversely associated with inflammation. Therefore, regulating the composition and abundance of microbiota, especially the dominant flora is of great significance for EAP/CUMS treatment. Based on the above, the current study provides new evidence that disturbance of the prostate microbiota is the main cause of EAP/depression. However, the precise interaction between the microbiota changes and depression induced by EAP and depression remains unknown and requires further study.

An increasing number of studies have linked the microbiota with host metabolic phenotypes, including glucose and insulin homeostasis, and amino acid metabolism. Therefore, the association between the prostate microbiota and metabolic phenotypes was examined in the current study. A small number of metabolic pathways with significant differences [CODH-PWY (reductive acetyl coenzyme A pathway), P163-PWY (L-lysine fermentation to acetate and butanoate), PWY-7031 (protein N-glycosylation (bacterial)) and P164-PWY (purine nucleobases degradation I (anaerobic))] was found after analyzing the relationship between prostate microbiota and host metabolic phenotypes. At present, specific protein glycosylation patterns have been proposed as biological disease markers for Alzheimer’s disease, attention-deficit hyperactivity disorder, and autism spectrum disorders (Pivac et al., 2011; Zhang et al., 2020). For example, through *in situ* fluorescence imaging, Zhang et al (Zhang et al., 2020) indicated a significant decrease in glycosylation and phosphorylation levels in depressed mice. These finding indicated that PWY-7031 (protein N-glycosylation (bacterial)) is involved in the influence of prostate microbiota on brain function. Furthermore, previous reports have shown that astrocyte metabolism is increased in spreading depression, and the uptake of acetate and butyrate in cerebral cortex is also enhancement (Dienel et al., 2001). Therefore, the change of P163-PWY (L-lysine fermentation to acetate and butanoate) induced by prostate microbiota may contribute to the development of depression. The regulation of acetate and butanoate by acetyl coenzyme A further confirm the role of prostate microbiota in depression. Additionally, acetyl coenzyme A regulating depression through fatty acid metabolism also provided theoretical foundation for our finding. Interestingly, A large number of research demonstrated that purine metabolism is dysregulated in patients with major depressive disorder (Ali-Sisto et al., 2016; He et al., 2020), consistent with our findings, P164-PWY (purine nucleobases degradation I (anaerobic))] was different in three groups. These data provide mechanistic insights into how prostate microbiota controls brain function. *DCE29, Nocardioes, Helicobacter* and *Dorea* encoded these genes involved in these different metabolic pathways. Recently, a Tianjin Chronic Low-grade Systemic Inflammation and Health cohort study about the relationship between infection with *Helicobacter pylori* and depressive symptoms in the general population in China showed that *H pylori* infection was related to depressive symptoms in women in the general adult population (Gu et al., 2019). Therefore, not only the intervention of *Helicobacter* in the stomach, but also the intervention of *Helicobacter* in the prostate contribute to the prevention and treatment of prostatitis and depression. These finding expands the knowledge of prostate microbiota in modulating prostatitis complicated with depression.

There were some limitations to the current study, including that the study used very limited numbers of animals to explore prostatitis complicated with depression. Experiments employing a larger sample size are urgently needed to verify the association. In addition, further research is required to elucidate the pathways in which prostatitis complicated with depression and the material basis for mediating brain–prostate communication, thus laying the foundation for the prevention and treatment of psychological disorders.

In summary, this study characterizes, for the first time, the difference of exposure to EAP and CUMS on the prostatic microflora and metabolic profiles of rats. Our finding provides theoretical basis of the importance of the disorder of cell metabolism caused by microbiota in the pathogenesis of prostatitis complicated with depression, and supplements the potential mechanism of the microflora-prostate-brain axis in the occurrence and development of prostatitis complicated with depression. Based on the mechanism of this study, further studies will aim to determine the treatment of prostatitis by regulating the balance of microbiota.

## Data availability statement

The data presented in the study are deposited in the CNGB Sequence Archive (CNSA) of China National GeneBank DataBase (CNGBdb) with accession number CNP0003466.

## Ethics statement

The animal study was reviewed and approved by the Institutional Animal Care and Use Committee of Shanghai Jiao Tong University.

## Author contributions

Conception and design: FL; performance of the experiments: FL; collection and assembly of data: XX; data analysis and interpretation: ZW; final approval of manuscript: PW. All authors contributed to the article and approved the submitted version.

## Conflict of interest

The authors declare that the research was conducted in the absence of any commercial or financial relationships that could be construed as a potential conflict of interest.

## Publisher’s note

All claims expressed in this article are solely those of the authors and do not necessarily represent those of their affiliated organizations, or those of the publisher, the editors and the reviewers. Any product that may be evaluated in this article, or claim that may be made by its manufacturer, is not guaranteed or endorsed by the publisher.
